# Elimination of cells with local mismatch in differentiation timing contributes to synchronize tissue development

**DOI:** 10.1016/j.isci.2025.113135

**Published:** 2025-07-17

**Authors:** Maleaume Soulard, Diego Andrés Contreras, Bruno Monier, Thomas Mangeat, Vanessa Dougados, Jennifer Zanet, Francis Corson, Vincent Hakim, François Payre, Anne Pélissier-Monier

**Affiliations:** 1 Molecular Cellular and Developmental Biology (MCD), Centre de Biologie Intégrative (CBI), Université de Toulouse, CNRS, UPS, 31000 Toulouse, France; 2 Laboratoire de Physique de l’Ecole Normale Supérieure, CNRS, Ecole Normale Supérieure, PSL University, Sorbonne Université, Université Paris Cité, Paris, France; 3 LITC Core Facility, Centre de Biologie Intégrative, Université de Toulouse, CNRS, UPS, 31062 Toulouse, France

**Keywords:** Cell biology, Developmental biology

## Abstract

During animal development, cells communicate to ensure tissue-wide synchronization of differentiation. While several mechanisms contributing to cell coordination have been described, whether additional mechanisms are at play should cells locally desynchronize remains unknown. Here, we investigate the responses to experimentally induced desynchronized cells during *Drosophila* epidermis development. We report that cells that behave as if they were “too young” or “too old”, collectively referred to as heterochronous cells, sort out from their normally timed neighbors. Cell sorting is associated with alterations of junctions, cytoskeleton, and cell mechanics. Moreover, local heterochrony ultimately leads to cell elimination. Importantly, we find that some cells naturally undergo either premature or delayed differentiation during development and are similarly eliminated from the tissue. These results show that local imbalance in differentiation timing affects both cell interactions and mechanics, leading to cell sorting, and elimination as a way to correct local heterochrony and safeguard the synchrony of epithelium differentiation.

## Highlights

• Some cells accidentally loose differentiation synchrony along epidermal development
• Heterochronic cells remodel their junctions and mechanics, leading to cell sorting
• Acto-myosin is bilaterally enriched at the interface of cells with mismatched timing
• Heterochronic cells and their direct normal neighbors are eliminated by apoptosis

## Graphical abstract

**Figure:**
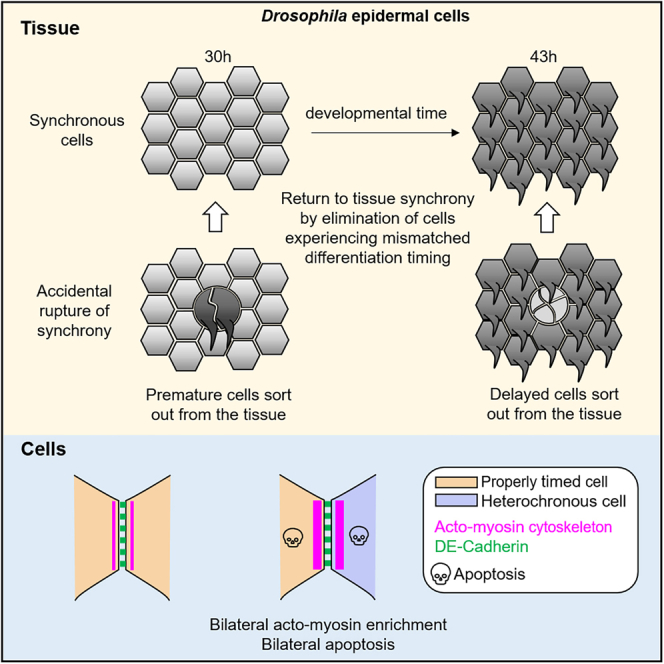

## Introduction

The development of animal embryos primarily relies on setting up specific spatiotemporal patterns of gene expression. While we have a wealth of information on how spatial patterns are progressively built during embryogenesis, the entire spectrum of mechanisms that ensure proper timing of tissue morphogenesis, growth, and differentiation remains to be fully elucidated. A key challenge for developing tissues is that all relevant cells adopt the same behavior, with the same pace, and therefore stay coordinated throughout the successive waves of differentiation. Many mechanisms ensuring cell coordination are known, which rely on juxtacrine, paracrine, or endocrine communications. Examples include Notch-Delta interactions for synchronization of presomitic mesoderm cells in vertebrates,^1^ small molecule exchanges via gap junctions for coordination of myogenic cells,^2^ or the diverse roles of the retinoic acid hormone during organogenesis.^3^ Nevertheless, it remains unclear how the tissue would react if in spite of these coordinating mechanisms some cells desynchronized and became more or less mature than their neighbors, creating local heterochrony. Is there any possibility for the tissue to detect, overcome, and ideally correct temporality defects?

The *Drosophila* epidermis is a fruitful model of morphogenesis and it appears well suited to address the importance of cell coordination during the progressive development of a tissue. In the *Drosophila* embryo, following three rounds of cell division, epidermal cells eventually undergo terminal differentiation from stage 15 onwards,^4^ when they engage prominent remodeling of their three-dimensional shape. These cell shape changes are characterized by the growth of apical actin-rich extensions referred to as trichomes, which will be reinforced by cuticle deposition. Trichome differentiation is governed by a transcription factor named Shavenbaby (Svb).^5^^,^^6^ Svb is specifically expressed in any cell that will make a trichome, but Svb is initially produced as a long-sized protein acting as a transcriptional repressor (Svb^REP^, 1354aa). Trichome formation is temporally regulated by small peptides called Polished rice (Pri), which are expressed in epidermal cells in response to a wave of the systemic hormone ecdysone at stage 15. Indeed, Pri peptides allow the Svb protein to undergo proteasome-dependent maturation, switching its function into a transcriptional activator (Svb^ACT^, 909aa) that triggers trichome differentiation.^7^^,^^8^^,^^9^ Svb^ACT^ directly activates the expression of a battery of target effector genes, e.g., encoding actin remodeling factors, or components of the apical extracellular matrix,^10^ such as ZP-domain proteins that organize apical sub-compartments to shape growing trichomes.^11^ Interestingly, the expression of Svb^REP^ coincides with the time window of the last rounds of epidermal cell division.^7^ The switch in Svb transcriptional activity is therefore associated with the temporal separation of proliferating vs*.* differentiating phases of epidermis development. However, how the epidermal tissue would react if some “young cells” (expressing Svb^REP^) became mixed with “older cells” (expressing Svb^ACT^) has not been investigated so far, since the embryonic epidermis suffers from significant limitations in terms of genetic manipulations. In addition, unlike earlier stages of ectoderm formation that have been extensively studied to understand cell dynamics during morphogenesis (e.g.,^12^^,^^13^^,^^14^^,^^15^), terminal differentiation of embryonic epidermal cells is far less accessible to imaging, in both fixed and live tissues due to cuticle deposition and muscle activity. Alternative systems are therefore needed to address the question of whether the tissue can manage local breaches in the timing of cell differentiation.

*Drosophila* is a holometabolous insect, meaning that most larval tissues formed during embryogenesis are eliminated by histolysis and new adult structures form during metamorphosis. This is the case for larval epidermal cells, which are removed through programmed cell death and replaced by newly formed adult epidermal cells. The dorsal thoracic epidermis, or notum, derives from the dorsal-most part of imaginal wing disc cells. These cells migrate from two contralateral wing discs and, 6 h after pupal formation (APF), converge and fuse dorsally in a way coordinated with larval epidermis removal.^16^ After thoracic fusion, waves of epidermal cell proliferation occur until around 30 h APF.^17^ The first sign of terminal differentiation occurs at around 39 h APF, when epidermal cells start to form robust actin-rich extensions, leading to the adult trichomes.^7^ Therefore, as during embryonic development, pupal epidermal cells first proliferate to yield the right number of cells, and then differentiate to acquire their function. Many works have well established the fly notum as a paradigm of epithelial morphogenesis, since it develops as a quasi-2D epithelium and is accessible to high-resolution imaging.^18^^,^^19^^,^^20^^,^^21^ In addition, recent work shows that adult trichome differentiation is also governed by Svb, in a molecular way similar to what occurs in the embryo.^7^ Finally, this tissue is also amenable to advanced genetic approaches, including generation of a mosaic tissue.^22^ These properties make the notum epidermis an attractive model to study the temporal control of cell differentiation.

Here, we investigate the consequences of local desynchronization of cell differentiation within the developing pupal notum. To address this question, we used a genetic mosaic strategy to express Svb repressor (Svb^REP^) or activator (Svb^ACT^) isoforms in clonal groups of cells, within otherwise normally developing epidermal tissue. We show that Svb^ACT^ is sufficient to induce premature differentiation, while Svb^REP^ prevents the ultimate step of differentiation, when compared to their neighbors. Our findings indicate that local heterochrony in differentiation leads to changes in cell proliferation, mechanical properties, and cell/cell interactions. They further suggest the existence of a surveillance mechanism that allows the tissue to detect, sort out, and ultimately eliminate heterochronous cells and their neighbors in order to synchronize epidermal differentiation. Finally, we provide evidence that naturally occurring heterochronous cells are also eliminated during pupal development.

## Results

### Generation of heterochronous cells in the pupal notum

**Figure 1:**
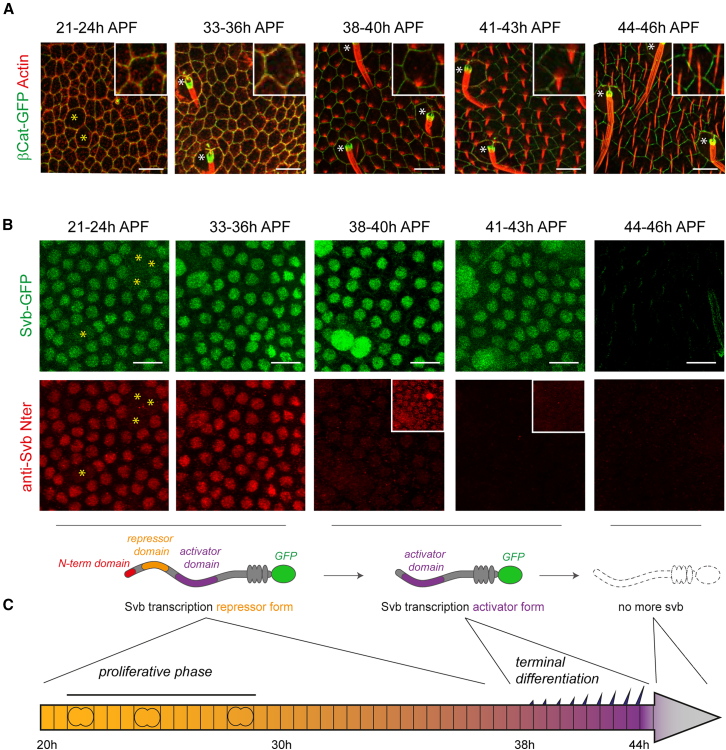
Timeline of cellular and molecular processes during pupal notum formation (A) Confocal microscopy images (projections) of control nota expressing *β-catenin::GFP* (green). Tissues were fixed at the indicated time points and stained for F-actin (red), which reveals progressive trichome differentiation. Closeups focusing on one cell are shown on the top right of each image. Yellow asterisks mark dividing epidermal cells, and white asterisks mark microchaetes, which are sensory organs not considered in this study. At least 5 individuals were observed *per* time point. (B) Confocal images of control nota expressing *svb::GFP* knock-in transgene (green). The C-terminal GFP tags both the full-length Svb^REP^ protein, as well as the N-terminally truncated protein Svb^ACT^ (schematized below). Tissues were fixed at the indicated time points and stained for Svb^REP^ (using an antibody against the N-terminal domain specific of Svb^REP^, in red). The plane of the nuclei was identified thanks to DAPI staining (not shown). Cells marked with yellow asterisks are those dividing. Insets show the image with enhanced signal. 8 to 9 nota were imaged *per* time point. (C) Schematic timeline of cellular and molecular processes during pupal notum differentiation. Scale bars: 10 μm.

Epidermal cells of the *Drosophila* dorsal thorax, or notum, form a highly homogeneous population of cells that initially proliferate up to 30 h APF, and then engage into terminal differentiation at 38–39 h APF. Terminal differentiation, which is under the control of Svb, is characterized by the formation of trichomes that grow at a similar rate across the tissue until approximately 45 h APF (Figures 1A and 1C).

We monitored the expression of all Svb protein molecules -*i.e.* non-processed and processed forms-, thanks to a *svb::GFP* knock-in line^23^ (green signal on Figure 1B). In addition, we also specifically detected the repressor form of Svb thanks to an antibody raised against the N terminal region of Svb^REP^ (red signal on Figures 1B and 1C),^8^ which is degraded upon the processing into Svb^ACT^. Figure S1A illustrates the specificity of the tools that we used to detect either Svb^REP^ or all Svb isoforms, as revealed by signal loss following Svb knockdown upon *svb* RNAi expression. Along notum development, we observed a strong accumulation of Svb^REP^ when epidermal cells divided, from L3 stages (Figure S1A) to around 30 h APF (Figures 1B and 1C). Svb^REP^ is maintained until 38–40 h APF (Figures 1B and 1C), at which point its levels dramatically decrease, coherently with the timing of *pri* expression.^7^ Since the total levels of Svb proteins (visualized using Svb:GFP) stay unchanged at 38–40 h APF, we conclude that most Svb molecules switch into the activator form (Figures 1B and 1C), which triggers terminal differentiation (Figures 1A and 1C). Finally, Svb was no longer detected at around 44–45 h APF, when cells have fulfilled their terminal differentiation and trichomes have reached their final size (Figures 1A–1C). Together with previous works,^7^^,^^17^ these data thus provide a detailed temporal framework to address the putative impact of cell heterochrony.

We then sought to generate groups of desynchronized cells, either “young” cells among “old” cells or the reciprocal. For this purpose, we took advantage of germinal isoforms of Svb that act either as the long repressive form (isoform OvoA, named hereafter Svb^REP^), or as the shorter activator form (isoform OvoB, named hereafter Svb^ACT^), both insensitive to Pri peptides.^8^ We expressed one or the other Svb isoform using a random *flp-out* clonal approach^24^ leading to mosaic epidermal tissues.

**Figure 2:**
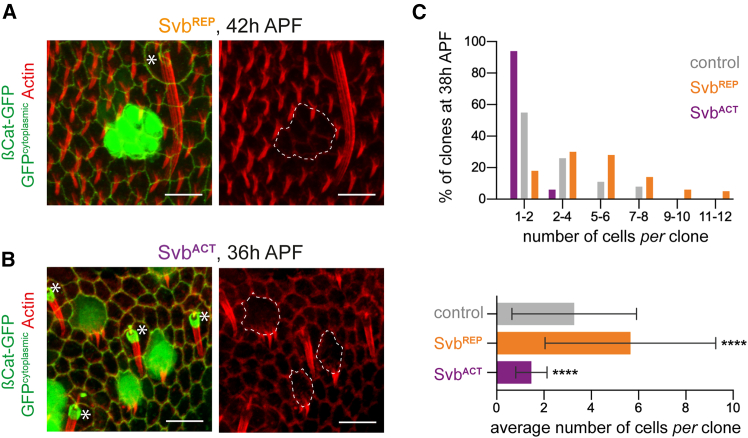
Generation of local heterochrony in the developing pupal notum (A) A clone of epidermal cells expressing Svb^REP^ in the pupal notum (GFP-positive, green cytoplasm) does not form trichomes (F-actin, red) at 42 h APF, contrary to other epidermal cells of the tissue. Cell contours are revealed by β-catenin-GFP (green junctions). The asterisk is at the base of a sensory microchaete. Clone outlines are highlighted by dashed lines. 5 nota/22 clones observed. (B) Cells of clones expressing Svb^ACT^ in the pupal notum (GFP-positive, green cytoplasm) extend trichomes (F-actin, red) at 36 h APF contrary to all other epidermal cells of the tissue. Cell contours are revealed by β-catenin-GFP (green). Asterisks indicate microchaetes. Clone contours are highlighted by dashed lines. 5 nota/35 clones observed. (C) Histogram (top) showing the distribution of clones according to their number of cells in controls (expressing RFP), Svb^ACT^ or Svb^REP^ clones. 7 nota with 86 clones, 11 nota with 85 clones, and 7 nota with 65 clones were observed for control, Svb^REP^ and Svb^ACT^ conditions, respectively. Bottom: data are represented as mean +/− standard deviation. Statistics: Kruskal-Wallis tests comparing Svb^ACT^ or Svb^REP^ to controls; *p* value: ∗∗∗∗, <0.0001. Scale bars: 10 μm.

Mosaic expression of Svb^REP^ led to clonal cells that do not differentiate trichomes, while surrounding cells developed normally and form robust actin-rich trichomes at 42 h APF (Figure 2A). It is currently unknown whether these Svb^REP^ cells could eventually differentiate with a delay, yet they form “younger” undifferentiated cells among more mature differentiated cells. Reciprocally, mosaic expression of Svb^ACT^ was sufficient to force premature trichome formation as soon as 30 h APF, while neighboring cells had not begun to change their shape (Figure 2B). Of note, multiple trichomes *per* cell could also be observed in this type of clone, similarly as reported in the pupal wing,^5^ possibly reflecting higher expression levels of Svb^ACT^ than normally reached by endogenously processed Svb. In addition to actin, premature trichomes (whether unique or multiple) appeared similar to normally timed trichomes, since they also comprised direct trichome effectors, such as Forked or Dusky-like (Figure S1B). These factors characterize the differentiation stage as: (1) they are not expressed before trichome formation, (2) their transcription is directly activated by Svb^ACT^, and (3) they directly contribute to trichome morphogenesis in the embryo and in the adult.^11^^,^^25^

When compared to control clones that express only RFP, we noticed that Svb^REP^ clones consistently contained a larger number of cells, as shown by both the distribution of clone sizes and the average number of cells *per* clone (Figure 2C). These enlarged clones suggest that Svb^REP^ cells have increased proliferative capabilities, which is consistent with cells remaining in a “young state”. Reciprocally, there are less cells in Svb^ACT^ clones (“old state”) than in controls (Figure 2C), which could be due, at least in part, to decreased proliferation.

Altogether, based on three criteria (formation of trichomes, expression of differentiation genes, and proliferative capacity), these results validate our approach to generate groups of desynchronized cells in the notum epidermis: either young cells (Svb^REP^ cells) among older neighbor cells, or old cells (Svb^ACT^ cells) among younger neighbor cells.

### Heterochrony induces cell sorting in the pupal notum

**Figure 3:**
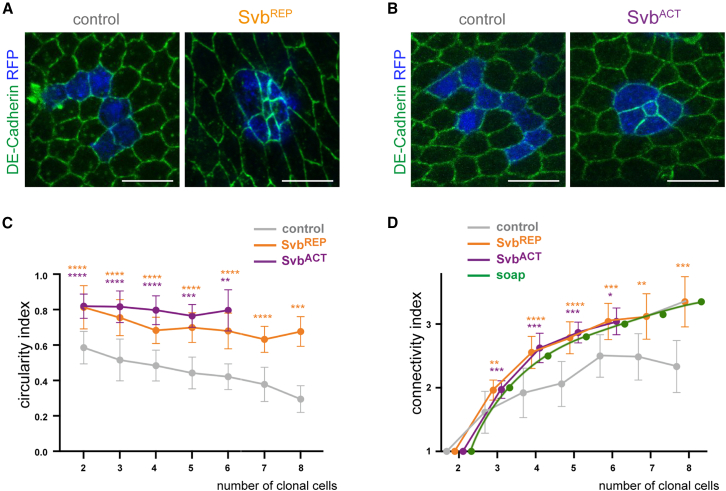
Heterochronous cells sort from properly timed cells (A) At 38 h APF, clonal Svb^REP^ cells in the notum (RFP-positive cells, blue) group together, contrary to control clonal cells. This is particularly obvious in the apical plane when looking at cell contours (revealed by DE-cadherin, green). (B) At 20 h APF, clonal cells expressing Svb^ACT^ in the pupal notum (RFP-positive cells, blue) also group together as opposed to controls. Cell contours were stained by DE-cadherin (green). (C) Graph plotting the clone circularity index against the number of clonal cells, in control (gray), Svb^REP^ (orange), or Svb^ACT^ (purple) clones. For controls, 20/13/22/10/11/10/6 clones were counted with respectively 2, 3, 4, 5, 6, 7, and 8 cells. For Svb^REP^, 12/13/22/12/12/8/10 clones were counted with respectively 2, 3, 4, 5, 6, 7, and 8 cells. For Svb^ACT^, 32/20/7/6/3 clones were counted with respectively 2, 3, 4, 5, and 6 cells. (D) Graph showing the connectivity index of clones plotted against the number of clonal cells, in control, Svb^ACT^ or Svb^REP^ clones. The theoretical behavior (green) is based on soap bubbles, an iconic physical model for the minimization of contacts.^26^ For the control context, 13/22/10/11/10/6 clones were counted with 3, 4, 5, 6, 7, and 8 cells, respectively. For Svb^REP^ context, 18/32/13/12/11/10 clones were counted with, respectively 3, 4, 5, 6, 7, and 8 cells. For Svb^ACT^ context, 24/8/6/4 clones were counted with, respectively 3, 4, 5, and 6 cells. In C and D, data are represented as mean +/− standard deviation. Statistics: Mann-Whitney tests comparing Svb^ACT^ or Svb^REP^ to controls; *p* value: ∗∗∗∗ <0.0001, ∗∗∗ <0.001, ∗∗ <0.01, ∗ <0.05. Scale bars: 10 μm.

We next investigated the consequences of local heterochrony for epidermal cells. Remarkably, the tissue responded to the presence of desynchronized cells by cell sorting. While control clones displayed a random shape, clones of delayed cells (Svb^REP^) featured a striking round shape (Figure 3A). The same behavior was observed in clones of premature cells (Svb^ACT^), which also displayed a round shape (Figure 3B). In each case, we calculated a circularity index, ranging from 0 to 1, 1 being the index of a perfect circle. This analysis confirms that both types of heterochronous cells group themselves in more circular clones than do control cells, whatever the number of cells *per* clone (Figure 3C).

If clones of heterochronous cells display a rounder shape than control clones, one would also expect less cell-mixing, namely that heterochronous cells have preferentially heterochronous cells as immediate neighbors. To check this property, we counted for each heterochronous cell the number of interactions with other heterochronous cells. We named “connectivity index” the average number of homotypic interactions (i.e., interactions between clonal cells, see Figure S2 for an illustration of how the connectivity index was measured). We compared the connectivity indexes of desynchronized clones to: (1) control clones; (2) theoretical clones with maximal connectivity, inspired by soap bubble analysis.^26^ As expected, the connectivity index increases with the clone size, as quantified in controls (Figure 3D). When compared to control clones of similar size, heterochronous cells (either too young or too old) displayed a clear increase in the connectivity index, reaching theoretical values of maximal connectivity (Figure 3D).

Together, these data indicate that epidermal cells experiencing either premature or postponed differentiation favor interaction between themselves, and minimize interactions with their wild-type neighbors. In other words, heterochronous cells sort out from properly timed cells.

### Altered mechanics as a cellular response to heterochrony

Cell sorting has previously been reported for mis-specified cells, i.e., cells with a modified fate due to alterations in signaling pathways or transcriptional networks.^27^^,^^28^^,^^29^^,^^30^^,^^31^^,^^32^^,^^33^^,^^34^^,^^35^^,^^36^^,^^37^^,^^38^^,^^39^ We therefore wondered whether similar mechanisms, involving alteration of cell-cell adhesion and of the cytoskeleton, were at play in clones of heterochronous cells.

**Figure 4:**
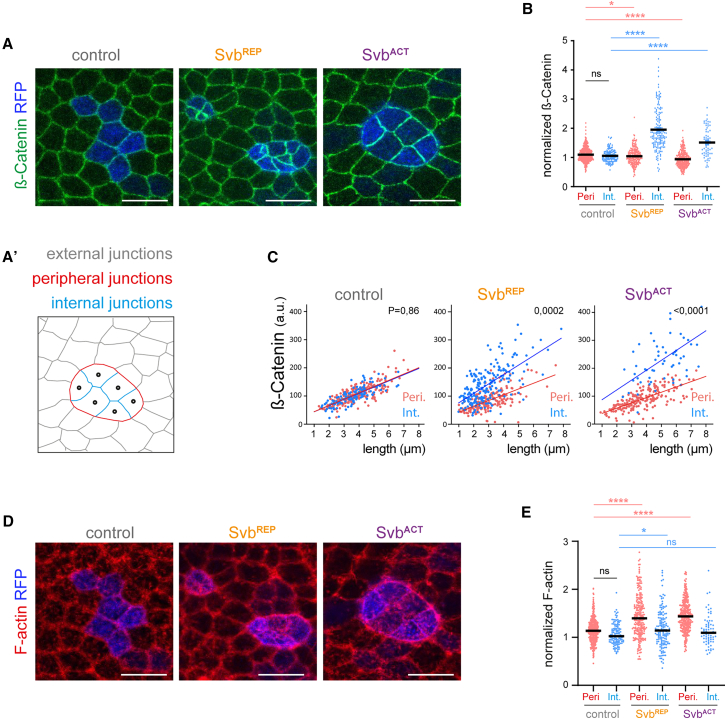
Heterochronous cells and their direct neighbors show altered junctions and cytoskeleton (A) Confocal images of control, Svb^REP^ or Svb^ACT^ clones at 38 h APF show the distribution of β-catenin (green), a major component of adherens junctions. Clonal RFP-positive cells appear in blue. (A′) Nomenclature used in Figures 4, 5, 6, and S3: internal junctions are homotypic junctions between two clonal cells (blue), peripheral junctions are heterotypic junctions between clonal and non-clonal cells (red), external junctions are homotypic junctions between two non-clonal cells (gray). (B) Quantifications of normalized β-catenin signal in control, Svb^REP^ and Svb^ACT^ clones, for each peripheral (red) or internal (blue) junction. 16 nota with 304 peripheral junctions and 132 internal junctions were quantified in controls; 8 nota with 231 peripheral junctions and 158 internal junctions in Svb^REP^ context; 20 nota with 282 peripheral junctions and 63 internal junctions in Svb^ACT^ context. (C) Graphs plot raw β-catenin intensity against the length of peripheral (red) and internal (blue) junctions, in control, Svb^REP^ or Svb^ACT^ clones. For controls: *n* = 225 peripheral and 121 internal junctions; for Svb^REP^: *n* = 195 peripheral and 209 internal junctions; for Svb^ACT^: *n* = 197 peripheral and 66 internal junctions. Each dot represents a value. Data were analyzed with simple linear regression by comparing slopes and intercepts using Prism. (D) Confocal images of control, Svb^REP^ or Svb^ACT^ clones at 38 h APF showing F-actin staining (red). Clonal RFP-positive cells appear in blue. The clones shown in (D) are the same than those shown in (A). (E) Quantifications of actin intensity, with 16 nota/165 peripheral junctions and 50 internal junctions in controls, 8 nota/244 peripheral junctions and 147 internal junctions in Svb^REP^ context, and 20 nota/314 peripheral junctions and 70 internal junctions in Svb^ACT^ context. In B and E, each value is represented by a dot, and black lines indicate medians. Statistics: Mann-Whitney tests; *p* value: ∗∗∗∗ <0.0001, ∗ <0.05, ns (non-significant) >0.05. Scale bars: 10 μm.

In order to understand how heterochronous cells minimize interactions with properly timed cells, we analyzed key components of adherens junctions (β-catenin, DE-cadherin) and of the acto-myosin cytoskeleton (polymerized Actin). We named “internal junction” a homotypic junction between two clonal cells, “peripheral junction” a heterotypic junction between a clonal cell and a wild type cell, and “external junction” a junction between two wild type cells (see Figure 4A’). Analysis of β-catenin and DE-cadherin distribution showed either no difference or only a slight decrease in peripheral junctions of heterochronous cells (Figures 4A, 4B, S3A, and S3B). In contrast, there was a high enrichment of β-catenin and DE-cadherin at internal junctions of both premature and delayed cells (Figures 4A, 4B, S3A, and S3B). This accumulation of junctional components in heterochronic internal junctions does not depend on the junction size (Figure 4C and S3C).

Actin changes displayed a reciprocal pattern, with only marginal increase (if any) of actin at internal junctions of heterochronous cells (Figures 4D and 4E). On the other hand, peripheral junctions were characterized by strong accumulation of F-actin forming a prominent “cable” all around clones of “young cells” in a late tissue, or of “old cells” in a young tissue (Figures 4D and 4E).

**Figure 5:**
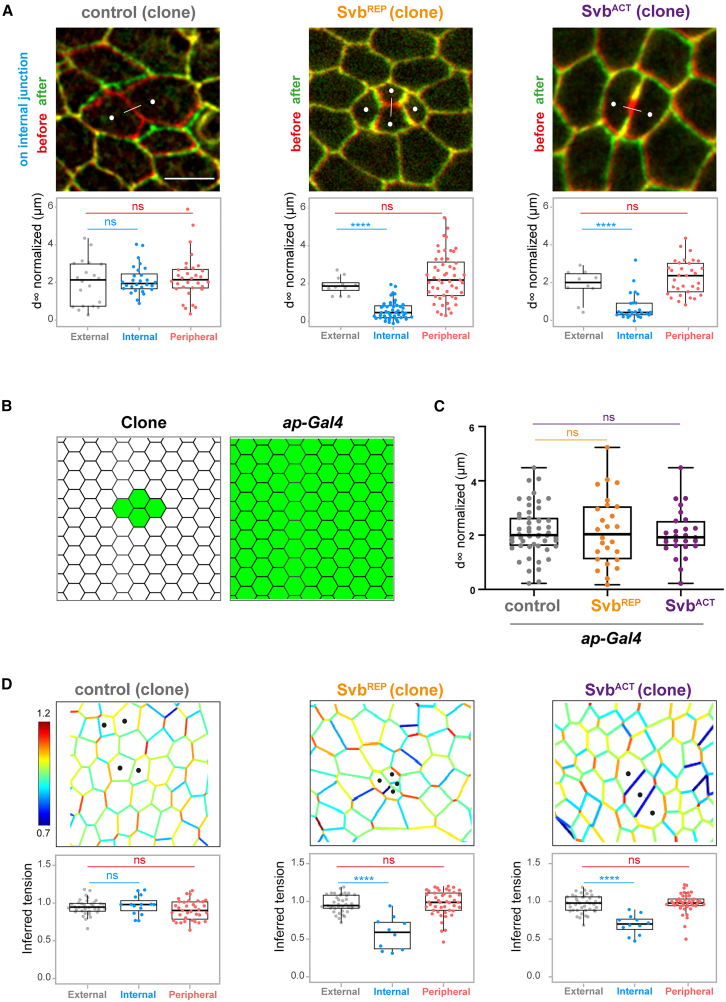
Altered mechanics as a cellular response to heterochrony (A) Top: examples of laser ablation experiments of internal junctions of control, Svb^REP^ or Svb^ACT^ clones. Pictures show the merge of two images, one taken just before the ablation (red) and the other at final recoil (green). Dots show clonal cells; lines indicate the ablation site. Scale bar: 10 μm. Bottom: boxplots showing the final displacement at t = ∞ after ablation of external, internal or peripheral junctions, in control, Svb^REP^ or Svb^ACT^ clones. 13 nota/20 junctions, 8 nota/28 junctions and 15 nota/32 junctions were analyzed for respectively external, internal and peripheral junctions in controls; 11 nota/12 junctions, 16 nota/48 junctions and 14 nota/52 junctions analyzed for, respectively, external, internal and peripheral junctions in Svb^REP^ context; 7 nota/10 junctions, 13 nota/28 junctions and 13 nota/33 junctions analyzed for, respectively, external, internal and peripheral junctions in Svb^ACT^ context. (B) Drawings schematize the two approaches used to express Svb^REP^ and Svb^ACT^, i.e., either mosaic expression (clone) or homogeneous expression (*ap-Gal4*). (C) Boxplots showing the final displacement at t = ∞ after photoablation at the level of junctions, in nota expressing RNAi-*luc* (control), Svb^REP^ or Svb^ACT^ driven by *ap-Gal4*. *N* = 51 junctions for controls, 26 for Svb^REP^ and 27 for Svb^ACT^. *p* values were computed using Kruskal-Wallis tests, and were >0.05 (non-significant, ns) in all cases. (D) Top: images of tissues containing control, Svb^REP^ or Svb^ACT^ clones analyzed by force inference. Tension values (color-coded) were calculated from the analysis of segmented images. Dots show clonal cells. Bottom: boxplots showing inferred tensions in external, internal or peripheral junctions in control, Svb^REP^ or Svb^ACT^ clones. 36/15/35 junctions were analyzed for external, internal and peripheral junctions in controls; 45/10/39 junctions for external, internal and peripheral junctions in Svb^REP^ context; 45/12/45 junctions analyzed for external, internal and peripheral junctions in Svb^ACT^ context. In boxplots, center lines show the medians; box limits indicate the 25th and 75th percentiles as determined by R software; each value is represented by a dot. Whiskers extend 1.5 times the interquartile range from the 25th and 75th percentiles in (A and D), and extend from minimum to maximum in (C). Statistics: Wilcoxon-Mann-Whitney test (A) or randomized test (D). *p* values: *p* < 0.0001, ∗∗∗∗; ns, non-significant.

These changes in the distribution of cytoskeleton and junction components suggested that heterochronous cells might have altered mechanical properties. Therefore, we explored this hypothesis through the analysis of mechanical features, in particular for internal homotypic and peripheral heterotypic junctions that displayed most prominent defects in molecular composition. First, we performed laser ablation on the different types of junctions, in the three genetic contexts (control, Svb^ACT^ and Svb^REP^ clones), and analyzed the final recoil in each case (Figure 5A). Internal and peripheral junctions of control clones behaved similarly to external junctions in all conditions (Figure 5A), confirming that the experimental manipulations used to generate mosaic clones do not impinge *per se* on cell properties. In contrast, and as expected from the accumulation of DE-cadherin and β-catenin (and the observation of associated zigzag junctions Figure S3A), internal junctions of heterochronous clones exhibited a strongly reduced recoil velocity (Figure 5A), indicative of a reduced junction tension. Because there was no significant changes in the recoil velocity when Svb^ACT^ or Svb^REP^ were homogeneously expressed in the notum (Figures 5B and 5C), our results imply that the direct interaction of cells displaying unmatched differentiation timing acts as a local signal, which translates into changes in mechanical properties.

Despite strong actin enrichment (Figures 4D and 4E), Svb^REP^ or Svb^ACT^ peripheral junctions did not exhibit significant modification in recoil velocity compared to control junctions (Figure 5A). Hence, these results suggested that the junctions at the periphery of heterochronous clones are not subjected to higher tension than controls.

To complete the analysis performed by photoablation, we performed force-inference (Figure 5D), a method to estimate local tension of all junctions of a sample based on image analysis.^40^^,^^41^ Briefly, when every junction of a cell exhibits equivalent tension, all junctions have the same length, and angles between two adjacent junctions are very similar, e.g., 120° for a cell with six vertexes, leading to a regular hexagonal shape. If one junction undergoes increased tension, it displays a reduced length, and angles measured at both vertexes connected by the junction have wider values. Therefore, force-inference leads to a global cartography of the junction tensions in the tissue. In control conditions, no particular pattern was observed; neither at the level of the clone, nor elsewhere in the tissue (Figure 5D). On the contrary, internal junctions of heterochronous cells clearly showed hallmarks of a reduced tension (Figure 5D). Interestingly, peripheral junctions did not display altered tension (Figure 5D) despite the observed accumulation of actin (Figures 4D and 4E). Therefore, force-inference results fully support the conclusions drawn from photoablation. Thus, alteration of junctional and cytoskeletal components in heterochronous cells would be translated into an alteration of cellular mechanics, especially with a drop in internal junction tension.

### Myosin II is enriched but not activated at the clone boundary

**Figure 6:**
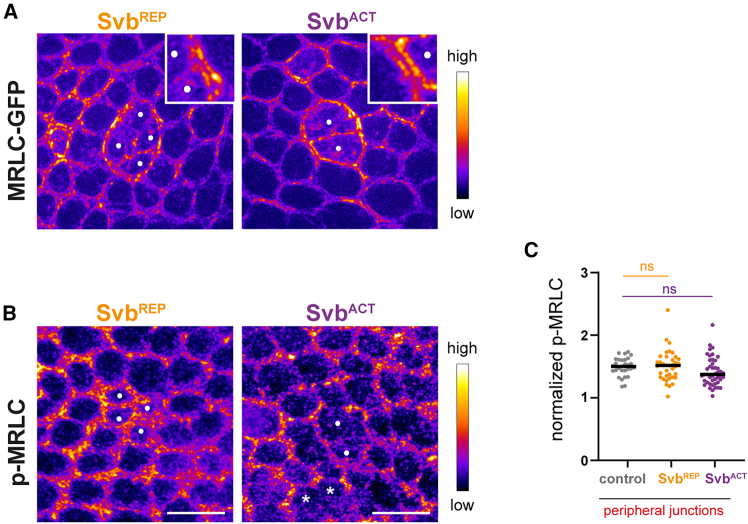
Myosin II is enriched but not activated at the clone boundary (A) Images of Svb^REP^ or Svb^ACT^ clones showing the distribution of non-muscular myosin II regulatory light chain, as seen using a *MRLC::GFP* strain and RIM super-resolution microscopy. Close-ups on clone boundaries are shown in insets. (B) Confocal images of Svb^REP^ or Svb^ACT^ clones show the distribution of phospho-MRLC, a readout of myosin activation. In A and B, intensity is color-coded, and clonal cells are indicated by dots or asterisks. Scale bars: 10 μm. (C) Quantification of fluorescence intensities measured on phospho-MRLC staining at the level of peripheral junctions of the clone in control, Svb^REP^ or Svb^ACT^ context. Each value is represented by a dot and medians are represented by black lines; Statistics: Mann-Whitney tests; *p* value: ns (non-significant) >0.05. 3 nota/30 junctions in control context; 7 nota/37 junctions in Svb^REP^ context; 10 nota/47 junctions in Svb^ACT^ context.

Actin enrichment is often associated with the enrichment of the molecular motor myosin II. As myosin II is a force generator, an increase in its levels at adherens junctions usually leads to increased tension.^42^ Since we observed high levels of actin but no increased tension at the boundary of heterochronous cells, we investigated the pattern of myosin II. Myosin II is composed by light and heavy chains, and its molecular motor activity requires the phosphorylation of the myosin regulatory light chain (MRLC).^42^ When using *MRLC::GFP*, we observed a strong accumulation at peripheral junctions of heterochronous cells (Figure 6A). However, the distribution of phospho-MRLC, a proxy of myosin activation, did not show significant increase at the clone border (Figures 6B and 6C). This result is consistent with the observation that tension is not increased at borders of mature heterochronous clones. We cannot exclude that, in earlier steps of the response to heterochrony, actomyosin accumulation could act as a conventional force generator to minimize contacts, leading to round clones. However, we propose that in borders of mature clones, it could act as a crosslinker (as previously documented^43^^,^^44^), possibly leading to rigidification of the cell boundary.

### Heterochrony promotes cell elimination of both properly timed and heterochronous cells

Cells whose fate is mis-specified reorganize their cytoskeleton to minimize contacts with their surroundings,^27^^,^^28^^,^^34^ and they are also more prone to be eliminated by programmed cell death, in particular apoptosis.^27^ It has been suggested that cell sorting would be a first step to eliminate aberrantly specified cells.^34^ Does segregation of cells that have a correct cellular identity (epidermal fate) but are temporally shifted (premature or delayed differentiation) also lead to cell elimination?

**Figure 7:**
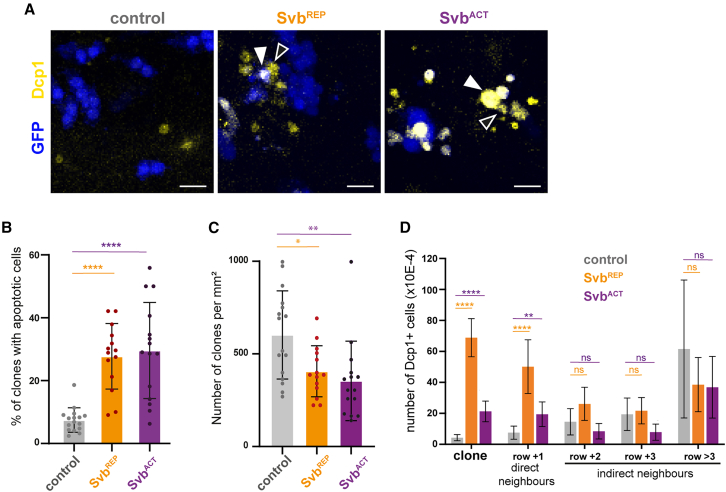
Heterochrony promotes cell elimination of both properly timed and heterochronous cells (A) Confocal images of control, Svb^REP^ or Svb^ACT^ clones stained with GFP (blue, clonal cells) and Dcp1 (yellow, apoptotic cells). White arrowheads point to some apoptotic cells within the clone, while empty arrowheads point to some non-clonal apoptotic cells that are in direct contact with clonal cells. Scale bar: 10 μm. (B) Graph showing the percentage of clones (GFP+ cells) containing at least one apoptotic cell (Dcp1+ cell) in the three conditions. Clones were analyzed on 16/14/15 nota in control, Svb^REP^ and Svb^ACT^ conditions, with 822, 485, and 453 clonal cells, respectively. (C) Graph showing the density of clones (number of clones normalized *per* mm^2^), in the same samples as (B). (D) Graph showing normalized number of Dcp1 positive cells in control, Svb^REP^ or Svb^ACT^ conditions, according to their distance from the clone: clonal cells (clone), cells which are direct neighbors (i.e., at the clone contact, row +1) or cells which are separated from the nearest clone by one (row +2), two (row +3) or more cell diameters (row >3). Data are from 7 nota with 231 clonal cells and 321 Dcp1+ cells in controls, 7 nota with 1536 clonal and 463 Dcp1+ cells in Svb^REP^, 7 nota with 324 clonal and 306 Dcp1+ cells in Svb^ACT^ conditions. In (B), (C), and (D), data are represented as mean +/− standard deviations and individual values are shown in (B) and (C). Statistics: Mann-Whitney tests (B) or t tests (C and D). *p* value: <0.05, ∗; *p* < 0.01, ∗∗; *p* < 0.0001, ∗∗∗∗; ns (non-significant) >0.05.

To answer this question, we examined whether desynchronized cells were more prone to cell death. First, we stained control and heterochronous clones with an antibody against the active form of the Dcp-1 caspase, which is an early marker of apoptosis^45^^,^^46^ (Figure 7A). Quantifications showed that there were significantly more heterochronous clones containing at least one apoptotic cell compared to controls (Figure 7B).

Since apoptosis is increased in clones of heterochronous cells, we reasoned that the number of heterochronous clones *per* sample notum might also be reduced compared to control clones. Accordingly, we found that the density of clones observed in samples at 40 h APF is smaller for Svb^REP^ or Svb^ACT^ clones, by comparison with control clones (Figure 7C). Moreover, analysis of the number of cells in Svb^ACT^ clones over time, between 20 h and 38 h APF, corroborated this result. Indeed, contrary to control conditions where the distribution of clones’ size was stable over time (Figure S4A), the distribution was unambiguously shifted to smaller clones over time following Svb^ACT^ expression (Figure S4A’’). However, we did not detect a reduction in the size of non-eliminated Svb^REP^ clones over time (Figure S4A’), likely due to the fact that cell death and proliferation are increased simultaneously. The reduction in the size of premature clones over time suggests that the tissue progressively eliminates heterochronous cells (when over-proliferation cannot compensate their loss, as is the case with expression of Svb^REP^). These observations show that it is important for cells to respect the temporal progression, at the risk of dying.

This suggested the existence of a safeguard mechanism that quickly eliminates heterochronous cells when they appear. It has been shown that when cells with aberrant fate appear in the larval wing disc, a sensing mechanism that involves acto-myosin recruitment at the periphery of the clone and JNK pathway activation leads to the elimination of mis-specified and neighboring cells.^27^^,^^34^ In the same vein, we observed using super-resolution random illumination microscopy (RIM) imaging^47^ that both heterochronous clonal cells and their wild-type neighbors contribute to the enrichment of myosin II at the clone boundary (Figure 6A). This result suggested that not only heterochronous cells react to the temporality defect, but also the neighboring cells in a non-autonomous way. To test whether both types of cells had an increased probability to die, we quantified the number of Dcp1 positive cells according to their distance from the clone (Figure 7D). We also compared the distribution of apoptotic cells observed in each genotype to the values expected from a model of random distribution (Figure S4B). We found that immediate neighbor cells (i.e., those sharing a junction with heterochronous cells, “+1”) are also significantly more prone to die, as opposed to wild-type cells located close to the clone but not in direct contact with desynchronized cells (separated only by one, two or three cell diameters, respectively “+2”, “+3”, and “>3”). While the same behavior was observed within and near Svb^REP^ and Svb^ACT^ clones, the higher number of dying cells detected upon Svb^REP^ expression (Figure 7D) likely reflects the increased number of clonal cells in this context (see also Figure 2C).

The behavior of untimely differentiating cells shares many features with that of mis-specified cells in the wing disc,^27^^,^^34^ raising the question of whether the correction of heterochrony involved the same signalling mechanism. To investigate this possibility, we then assayed whether the JNK pathway was activated in heterochronous cells and/or their immediate neighbors in the notum. We did not detect ectopic JNK activity neither in Svb^ACT^ and Svb^REP^ clones, nor in the surrounding tissue (Figure S5). Further work will be required to determine whether different types of cell mismatches (fate vs*.* timing) could lead to cell elimination via distinct signaling pathways. Alternatively, it is possible that the response to cell mismatches (fate and timing) might differ from one tissue to another.

### Correction of temporally mismatched differentiation during development

**Figure 8:**
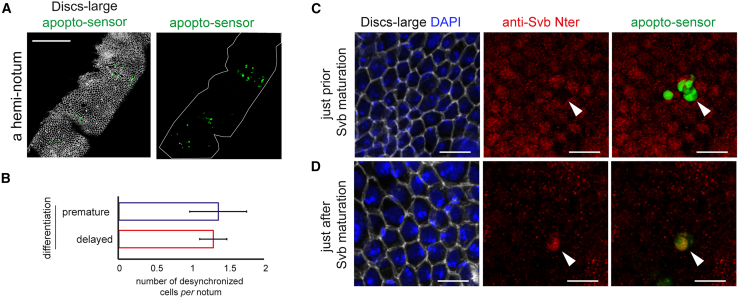
Correction of temporally mismatched differentiation during development (A) Low magnification confocal image of an entire hemi-notum stained for Discs-large to reveal the overall morphology, and expressing a GFP apopto-sensor, illustrating the proportion of cells dying at 38–39 h APF in the *ap-Gal4* domain. Scale bar: 100 μm. (B) Quantification of the number of desynchronized cells *per* notum, either premature (*n* = 19 cells, 14 nota) or delayed in their differentiation (*n* = 40 cells, 31 nota). Data are represented as mean ± SEM. (C and D) Confocal images taken at 38–39 h APF (the average timing of Svb processing) showing a notum in which cells have not processed Svb yet (C), or a notum in which cells have already processed Svb (D). Discs-large and DAPI staining reveal cell contours and nuclei; anti-Svb N-term and apopto-sensor reveal the presence of Svb^REP^ and apoptotic cells, respectively. Arrowheads point to naturally desynchronized cells, either a cell that has processed Svb prematurely (C), or a cell which is delayed as it has not processed Svb on time (D). Note that both types of desynchronized cells are apoptotic, the cell in C having started to fragment. Scale bars in (C and D): 10 μm.

Finally, we explored whether untimely differentiation and its correction by apoptosis naturally occur during notum epidermal development. To do so, we examined the differentiation timing of normal epidermal cells, i.e., in the absence of mosaic clones, and otherwise wild-type background but expressing the sensor of activated caspases GC3Ai^45^ to detect apoptosis. Since the Svb repressor is normally processed into the activator form in a rapid and synchronized manner (Figures 1B and 1C), we analyzed a large number of cells in the time window when Svb^REP^ to Svb^ACT^ processing occurs (38–39 h APF, Figure 8A). Using the antibody specific to the Svb^REP^ isoform (see Figure 1), we observed a small proportion (from 0 to 5 cells *per* notum) of cells that have already lost Svb^REP^ signal, while all neighbors have not yet accomplished Svb processing (Figures 8B and 8C). These cells therefore displayed a premature differentiation timing when compared to their neighbors. We also observed a similar proportion of cells that displayed delayed differentiation (from 0 to 4 cells *per* notum), since they were still positive to anti-Svb^REP^ staining at a stage where Svb has been processed throughout the tissue (Figures 8B and 8D). Importantly, we found that all naturally desynchronized cells observed (*n* = 59 cells, 45 nota), either slightly too old or too young, were eliminated from the tissue through apoptosis since they activated the apopto-sensor and became fragmented (Figures 8C and 8D). In contrast, in more than 99% of apoptotic cells, the status of Svb (unprocessed vs*.* processed) was similar to the status of Svb in the surrounding tissue (Figure S6), strongly supporting that apoptosis is not the cause but the consequence of untimely differentiation.

Therefore, despite the existence of different mechanisms to coordinate cell differentiation, some cells escape the temporal control of differentiation. Remarkably, they are detected and eliminated by apoptosis to safeguard the synchrony of tissue development.

## Discussion

Here, we investigated the importance of synchrony between differentiating cells in order to form a proper adult tissue, exploring the multiscale consequences of mingling between “early” (more proliferative) and “late” (undergoing terminal differentiation) cells. This was done by expressing the transcription factor Svb, whose activity does not lead to misspecification (as cells express normal genes for their lineage) but which was engineered to bypass the temporal control of its activity. Our findings show that untimely differentiation affects both the intrinsic properties and mechanics of heterochronous cells, as well as the behavior of their direct “normal” neighbors. The data further show the existence of a safeguard mechanism acting to correct local heterochrony, when it appears in the tissue. As recently reported for mis-specified cells, that are also sorted out and eliminated in the absence of fitness differences,^34^ heterechronous cells may be sorted out not because they lose in cell competition (Svb^REP^ cells would even display characteristics of “winner cells”), but because they are “not normal”. Therefore, sorting out abnormally timed cells could be a first step, before elimination, to ultimately ensure the synchronicity of differentiation in order to ensure the robustness of tissue development.

While our results show that cells are able to detect a difference in the timing of differentiation, the underlying mechanisms are still unknown. In the wing disc, it has been recently found that identification of cells with an abnormal fate relies on a cell surface code: fate-patterning pathways lead to the expression of a combination of cell surface proteins such as Robos, Teneurins or LRR (leucine rich repeats) proteins, unique to each cell fate.^28^ Any fate defect is thus reflected by an altered surface code, and apposition of cells with different surface codes is sufficient to activate interface surveillance. Further studies will be necessary to test the possibility that heterochronous cells are also detected by a similar mechanism, relying on an altered surface code. This hypothesis would imply that the surface code of each cell changes along the successive steps of epidermal differentiation, which remains to be demonstrated. Another possible mechanism for cells to detect heterochrony might be based on mechanics sensitivity. Indeed, it has been reported that acto-myosin-dependent tension progressively increases in the fly notum from 12 h to 30 h APF.^48^ In addition, differential actomyosin-dependent cell-cortex tension can mediate cell sorting in the early zebrafish embryo,^49^ as we observed in temporally mismatched notum cells. We therefore propose a model in which the tissue detects mismatches in differentiation timing based on local differential tension.

After the tissue has detected a mismatch in differentiation timing, temporally mismatched cells are eventually eliminated by apoptosis. Importantly, our findings show that both the heterochronic cells and their neighbors are more prone to die. This mechanism allows proper correction of accidental defects in timing synchrony without knowing which cell is altered and which one is normal, and ultimately ensures the elimination of sporadic heterochronous cells or small groups of cells. On the other hand, this correction mechanism would only have a marginal activity against a change in the development timing of a whole tissue or large fields of cells, as it might occur during evolution that involves heterochrony.

A feature shared with cells experiencing mis-specification is the strong accumulation of acto-myosin that drives cell sorting. Could the cytoskeletal cable forming at the interface between mismatched cells be a mandatory intermediate between the detection system and activation of the apoptosis cascade? This hypothesis is supported by a recent study showing that alteration of the acto-myosin cortex is sufficient to drive apoptosis in developing and tumoral tissues.^50^ If the cable proves mandatory for activation of apoptosis in interface surveillance, it will be critical to identify the mechanisms involved, whether it eventually culminates in JNK activation (mis-specified cells) or not (temporally mismatched cells). Since alterations in the actin cortex can also activate caspases to drive cell remodeling, for example during neuronal pruning,^51^ identification of molecular links between the cytoskeleton and the activation of caspases should be of interest for the understanding of various processes ranging from development and neuronal dynamics to tumorigenesis.

### Limitations of the study

Future studies will be required to test the importance of such a temporal surveillance mechanism in other contexts. In *Drosophila*, it could be interesting to examine the possible impact of desynchronization in the eye disc, where clonal expression of molecules such as Dpp and Delta, or Hedgehog, can locally trigger premature differentiation.^52^^,^^53^ However, these diffusible molecules act non-autonomously, which will require more complex analyses of the cellular behaviors.

Moreover, it would be interesting to investigate similarities and possible differences between interface surveillance mechanisms due to mismatches in cell fate^27^^,^^28^^,^^34^ or in cell timing (this work). In a given tissue, would both defects be sensed and corrected by the same mechanisms? Or do they involve distinct and specific pathways?

## Resource availability

### Lead contact

Further information and requests for ressources should be directed and will be fulfilled by the lead contact, Anne Pélissier-Monier (anne.pelissier-monier@utoulouse.fr).

### Materials availability

This study did not generate new unique reagents.

### Data and code availability

• All data reported in this paper will be shared by the lead contact upon request.
• This paper does not report original code.
• Any additional information required to reanalyze the data reported in this work is available from the lead contact upon request.

## Acknowledgments

We are grateful to the Bloomington Drosophila Stock Center, Vienna Drosophila Resource Center, and Developmental Studies Hybridoma Bank for providing us with flies and antibodies. We thank B. Ronsin for his help concerning imaging and data analysis. We thank F. Bosveld and Y. Bellaïche for help and advices, and M. Suzanne for her generous support for the last stages of this study. We also thank C. Polesello for critical reading of the manuscript. This work was supported by the ANR Chrononet (14-CE11-0010-01) and by grants from the CONICYT, Beca Chile (doctoral scholarship for DAC) and from la Ligue Nationale Contre le Cancer (doctoral 4^th^ year fellowship for MS).

## Author contributions

A.P.-M., M.S., F.C., V.H., and F.P. designed the project. M.S., B.M., and A.P.-M. performed the biological experiments with the help of J.Z., M.S., V.D., B.M., and A.P.-M. performed the laser ablation experiments. D.A.C. under the supervision of V.H. and F.C., and T.M. performed force inference analyses. M.S. and T.M. performed the RIM experiments. M.S., B.M., A.P.-M., and F.P. analyzed the experiments with the help of V.D., T.M., D.A.C., F.C., and V.H. for physical experiments. A.P.-M., F.P., and B.M. performed the experiments and analyses required to revise the manuscript. F.P., F.C., and V.H. obtained funding. A.P.-M. wrote the original manuscript. All authors commented on the manuscript. All authors read and approved the final manuscript.

## Declaration of interests

The authors declare no competing interests.

## STAR★Methods

### Key resources table

**Table:** 

REAGENT or RESOURCE	SOURCE	IDENTIFIER
**Antibodies**		

Rabbit anti-Svb-1S (1:1000)	Zanet et al.^9^	N/A
Rat anti-E-Cad-c (1:100)	DSHB	DCAD2; RRID: AB_528120
Mouse anti-βCat (1:200)	DSHB	N27A1; RRID: AB_528089
Rat anti-Dusky-like (1:500)	Fernandes et al.^11^	RRID:AB_2567622
Rabbit anti-Forked (1:1000)	Guild et al.^54^	N/A
Rabbit anti-GFP (1:500)	Torrey Pines Biolabs	Cat# TP401 071519, RRID:AB_10013661
Rabbit anti-cleaved Dcp1 (1:200)	Cell Signaling Technologies	Cat#9578; RRID: AB_2721060
Rabbit anti-Phospho-MRLC (1:200)	Cell Signaling Technologies	Cat#3671; RRID:AB_330248
Mouse anti-Dlg1-c (1:200)	DSHB	4F3; RRID: AB_528203

**Chemicals, peptides, and recombinant proteins**		

Phalloidin-Rhodamine (1:500)	Fischer Scientific	Cat# R415
Vectashield medium with DAPI	Vector Laboratories	Cat# H-1200

**Experimental models: Organisms/strains**		

*D. melanogaster*: hs-flp; actin<y+<Gal4; UAS-GFP	Chanut-Delalande et al.^7^	N/A
*D. melanogaster*: UAS-SvbACT	Delon et al.^5^	N/A
*D. melanogaster*: UAS-SvbREP	Delon et al.^5^	N/A
*D. melanogaster*: UAS-GC3Ai	Schott et al.^45^	N/A
*D. melanogaster*: actin<CD2<Gal4; UAS-RFP	BDSC	RRID: BDSC_30558
*D. melanogaster*: β-Catenin::GFP	BDSC	RRID: BDSC_8555 and BDSC_8556
*D. melanogaster*: DE-Cadherin::GFP	BDSC	RRID: BDSC_60584
*D. melanogaster*: Sqh ::TagRFPt	Ambrosini et al.^55^	N/A
*D. melanogaster*: Sqh ::eGFP	Ambrosini et al.^55^	N/A
*D. melanogaster*: svb ::GFP	Markus et al.^23^	N/A
*D. melanogaster*: UAS-mRFP	BDSC	RRID: BDSC_30557
*D. melanogaster*: “apterous::Gal4”: y w; P{GawB}ap[md544]/CyO	BDSC	RRID: BDSC_3041
*D. melanogaster*: w; P{TRE-EGFP}attP16	BDSC	RRID: BDSC_59010
*D. melanogaster*: UAS-RNAi Svb	VDRC	RRID:Flybase_FBst0464178
*D. melanogaster*: tub-Gal80ts	BDSC	RRID: BDSC_7018
*D. melanogaster*: UAS-RNAi luc	BDSC	RRID: BDSC_35788
*D. melanogaster*: en-Gal4, UAS RFP	BDSC	RRID: BDSC_30557

**Software and algorithms**		

Prism 8	GraphPad	RRID: SCR_002798
Illustrator	Adobe	RRID: SCR_010279
Fiji	https://fiji.sc/	RRID: SCR_002285

**Other**		

LSM 880 with Airyscan Confocal Laser Scanning Microscope	Zeiss	RRID:SCR_020925
LSM 900 with Airyscan 2 Confocal Laser Scanning Microscope	Zeiss	RRID:SCR_022263
Zeiss LSM 710 Confocal Inverted Microscope	Zeiss	RRID:SCR_018063
Leica TCS SPE	Leica	RRID:SCR_002140

### Experimental model and study participant details

#### Breeding conditions for model animals

The animal model used here is *Drosophila melanogaster*, in a context of *in vivo* experiments. In order to respect ethic principles, adult animals were anesthetized with CO2 before manipulation. To avoid release of flies outside the laboratory, dead flies were frozen before throwing them. Stocks of living flies were conserved in incubators, either at 18 or 25 degrees to maintain the flies in optimal conditions. Experiments were performed in both males and females indifferently, during metamorphosis between 20h and 45h APF (exact stage is indicated in each experiment). Experiments were conducted at 25°C.

##### *Drosophila* stocks and genetics

For mosaic clones in the thorax, we used the FLP-out technique.^24^ For this purpose, flies carrying the driver line *hs-flp; actin < y + < Gal4; UAS–GFP* flies^7^ were crossed with *UAS* lines, and larvae heat-shocked for 8 min at 37°C, 8 h before puparium formation. UAS responder lines were: *UAS-RFP* (for control clones)*, UAS–svb*^*ACT*^ (also known as *UAS-ovoB*)^5^*; UAS–svb*^*REP*^ (also known as *UAS-ovoA*)^5^ and *UAS-GC3Ai* (“apopto-sensor”, *i.e.* tool to detect apoptotic cells).^45^ For the analysis of JNK activation, clones were induced using *actin < CD2 < Gal4; UAS–RFP* (BDSC_30558). Fluorescent reporter lines were *ß-Catenin::GFP* (BL_8555 and BL_8556) and *DE-Cadherin::GFP* (BL_60584) to visualize adherens junctions, and *Sqh::TagRFPt* or *Sqh::eGFP*^55^ to visualize Myosin. The *svb::GFP* knock-in line was described previously.^23^

For laser ablation, stocks described above were combined in order to cross *hs Flp; DE-Cadherin-GFP ; UAS–svb*^*ACT*^ or *UAS–svb*^*REP*^ or *UAS-RFP* flies to a *w ;;act5C*< *y* + < *Gal4, UAS-nlsRFP/TM6b* stock.

Additional stocks were *en-Gal4, UAS-mRFP* (BDSC_30557), *ap-Gal4* (BDSC_3041), *TRE-GFP* (JNK reporter, BDSC_59010), *UAS-RNAi Svb* (RNAi targeting the 1S region of the somatic isoforms, VDRC_41584), *tub-Gal80*^*ts*^ (BDSC_7018), *UAS-RNAi luc* (control for some laser ablation experiments, BDSC_35788).

### Method details

#### Antibodies and confocal imaging

White pupae were picked and staged for the appropriate time at 25°C. Pupal thoraces were dissected in PBS, fixed for 20 min in 4% paraformaldehyde and processed for immunostaining. Staining was carried out as previously described^11^ using Rabbit anti-Svb1S (1:1,000), rat anti-DE-cadherin (DCAD2 from DSHB, 1:100), mouse anti-Armadillo/β-Catenin (N2 7A1 from DSHB, 1:200), rat anti-Dusky-like (1:500), rabbit anti-Forked (1:1000), rabbit anti-GFP (Torrey Pines, 1/500), rabbit anti-cleaved Dcp-1 (#9578 from Cell signaling Technology, 1:200), rabbit anti-phospho-MRLC (#3671 Cell signaling Technology, 1:200), mouse anti-Discs-large (4F3 from DSHB, 1:200). AlexaFluor-488, -555 or -647 secondary antibodies (Molecular Probes) were diluted at 1:200. Staining of F-actin was achieved using TRITC-phalloidin (Sigma, 1:500). Samples were mounted in Vectashield containing DAPI (Vector laboratories) to counterstain nuclei.

Each mosaic tissue showed clones of mutant cells surrounded by wild-type neighbouring cells, providing internal controls. A typical experiment contains five to ten dissected samples, of proper stage and genotype. Data have been collected in at least three independent experiments. Samples were imaged with laser scanning confocal microscopes (Zeiss or Leica). Images were processed using the Fiji software.

#### Super-resolution RIM microscopy

Super-resolution imaging was performed by Random Illumination Microscopy (RIM), using an upgrade of the system and method described previously.^47^ Briefly, we used a dynamic speckle illumination system composed of a fibered laser (with a wavelength centred on 561nm for RFP) and an optical spatial modulator for generating random phases (QXGA, fourth dimensions). This dynamic speckle illumination was injected to an inverted microscope (TEi Nikon) equipped with a X60 magnification, 1.49 NA objective (CFI APO, NIKON) and a SCMOS camera (ORCA-Fusion, Hamamatsu). The acquisition software (INSCOPER SA) enabled recording a 3D stack (6 planes, with a Z step of 200nm) in only 6 s, under a low-photobleaching regime (1 W cm−2). The method used for 3D image reconstruction was described previously^47^ and is also available at GitHub (https://github.com/teamRIM/tutoRIM).

#### Segmentation of epithelial cells and 2D inference of mechanical forces

The Tissue Analyser software was used (Fiji plugin). Prior to each segmentation, a background noise reduction (rolling ball method, ImageJ) was applied, followed by Gaussian filtering. Images were segmented semi-automatically using the Watershed method with mask seeds, with manual correction of segmentation errors. Tables were used to extract the parameters of the cells and boundaries. Based on the segmentation data, a Bayesian force inference method was used, as described in.^56^ From the segmentation data, the boundary stresses were inferred. The minimising parameters μ were adapted to suit the tissues, generally less than 1. The code was then modified to extract the values of junction tension around user-defined zones of interest.

#### Photo-ablation experiments

Laser ablation experiments were performed using a pulsed Lined Q switch Yag double laser (wavelength 532nm, pulse length 0.4ns, repetition rate up to 7kHz, 7μJ/pulse) steered by a galvanometer-based laser scanning device (Ilas2, Roper Scientific), mounted on a Leica DMI6000B inversed microscope. The laser beam was focused through an oil-immersion lens of high numerical aperture (Plan-Apochromat ×100/0.7-1.4, from Leica). Photo-ablation was done in the focal plane at the middle of adherens junctions, following a line of 2 μm for 80ms at 100% laser power (10 iterations, with a thickness of 1). Cell junctions were visualized using *DE-Cadherin::GFP* and placed in the centre of the field to optimise reproducibility. The type of junctions (external, internal or peripheral) was determined, before ablation, using RFP imaging.

Live images were acquired on a wide-field microscope equipped with a CCD cooled (HQ2, Ropper Scientific SA) camera with a pixel size of 64.5 nm, using a FITC/GFP filter and HBO illumination, to limit photobleaching. Metamorph coupled to ILAS software (Ropper) controlled the laser and the microscope. Acquisition was performed every 1.5 second during 7.5 seconds before ablation, and over a period from 55.5 to 79.5 seconds after ablation.

Alternatively, for ablation using UAS expression in the whole tissue, Svb isoforms or a neutral transgene (RNAi luciferase) were expressed using *ap-Gal4, arm::GFP*; *tub-Gal80*^*ts*^*,* during 8h before examination. Ablations were performed on LSM 880 microscope as previously reported.^57^

### Quantification and statistical analysis

#### Statistical analysis

Statistics were usually performed in Prism 8 (Graphpad). N and *p*-values, as well as the statistical tests used, are indicated in the figure legends. The normality of data sets was determined in Prism. For data sets that follow a normal law, we used two-tailed unpaired t-tests. For data sets that do not follow a normal law, we performed Mann Whitney or Kruskal-Wallis tests. For multiple tests, individual *p*-values were from t-tests, using a False Discovery Rate approach (Q = 1%) and two stage step-up method of Benjamini, Krieger and Yekutieli (https://www.mathworks.com/matlabcentral/fileexchange/27423-two-stage-benjamini-krieger-yekutieli-fdr-procedure). For comparison of values of force inference, we used randomised tests (https://thenode.biologists.com/quantification-of-differences-as-alternative-for-p-values/).

#### Quantification of circularity and connectivity

Circularity of the clones was computed using ImageJ, following the formula: circularity index = $4\pi \frac{A}{P^{2}}$ , where A is the area and P the perimeter. A value of 1.0 indicates a perfect circle.

Connectivity was calculated by: i/ counting for each cell of a clone the number of direct interactions with other clonal cells; ii/ calculating the average number of homotypic interactions among all the cells of the clone (see Figure S2 for an illustration of how the connectivity index was measured).

#### Quantification of normalized intensity

To measure junctional enrichment of DE-Cadherin, ß-Catenin, F-actin or phospho-MRLC, fluorescence intensity was measured by tracing a line on individual junctions either within the clone (internal junctions), or at the boundary of the clone (peripheral junctions). Regular junctions far away from the clone were also measured in each notum as they provide an internal control (external junctions). Mean fluorescence intensity of internal and peripheral junctions was then normalised by the intensity of external junctions of the same notum.

#### Quantification of apoptosis

To quantify apoptosis, we stained apoptotic cells using anti-activated Dcp1 antibody and detected control, Svb^REP^ and Svb^ACT^ clones using RFP. The tissue was counterstained with DAPI (not shown). A cell was considered apoptotic when its nucleus was of similar size than one of a non-apoptotic cell. Dcp1 staining with no DAPI staining or small dots of DAPI staining were considered as debris. To estimate the expected number of Dcp1-positive cells, in and around the clones, we used a simple probabilistic model (random sampling without replacement). Probabilities were calculated using the following formula: $\left(X=k\right)=\left(\begin{array}{l} m\\ k \end{array}\right)\left(\begin{array}{l} N-m\\ n-k \end{array}\right)/\left(\begin{array}{l} N\\ n \end{array}\right)$ , where *N*= total number of cells *per* image (4100 cells, on average), *m*= number of cells of interest (clones, or the population of +1, +2, +3, >+3 neighbours), *n*= number of Dcp1+ cells, *k*= the number of cells of interest being positive for Dcp1. The value of *k* displaying highest probability was kept as the expected number of Dcp1+ cells, in each condition. The number of +1, +2 and +3 neighbour cells was approximated from values observed in regular hexagonal tiling, taking into account the average number of cells *per* clones in the three genetic conditions (control, Svb^REP^ and Svb^ACT^). To allow comparison between genotypes, percentages of Dcp1+ cells *per* image were normalized using the average total number of Dcp1+ cells in the three conditions.

#### Analysis of photoablation experiments

Energy relaxation was analysed by monitoring the time-dependent separation of the two vertices whose common link has been removed, d_t_, with respect to the equilibrium distance d0. This relaxation is very close to exponential with a plateau, and thus characterized by a single time scale. Hence, strain curves could be fitted using the following formula:

$$\frac{d_{t}-d_{0}}{l_{c}}=\frac{d_{\infty }-d_{0}}{l_{c}}\left(1-e^{\frac{-t-t_{0}}{\tau }}\right)$$

where *d* is the distance and τ is the relaxation time scale and *l*_*c*_ the junction length. This formula is the same than the one used for *in silico* ablations of different cell edges from a dynamic vertex model.^58^ There is a direct proportionality between the final displacement at t=∞ and the initial velocity at t=0. Due to the temporal resolution after the photoablation, we used statics from $\frac{d_{\infty }-d_{0}}{l_{c}}$, and τ.

The analyses were performed with the ImageJ software, using MtrackJ Plugin, as previously described.^59^
