# Supplementary material for: Elimination of cells with local mismatch in differentiation timing contributes to synchronize tissue development
Source: iScience. 2025 Jul 17;28(8):113135. doi: 10.1016/j.isci.2025.113135 (PMC12337790; doi:10.1016/j.isci.2025.113135)
Supplement: Document S1. Figures S1–S6 [file mmc1.pdf]

iScience, Volume *28*

Supplemental information

Elimination of cells with local mismatch in differentiation timing contributes to synchronize tissue development

Maleaume Soulard, Diego Andrés Contreras, Bruno Monier, Thomas Mangeat, Vanessa Dougados, Jennifer Zanet, Francis Corson, Vincent Hakim, François Payre, and Anne Pélissier-Monier

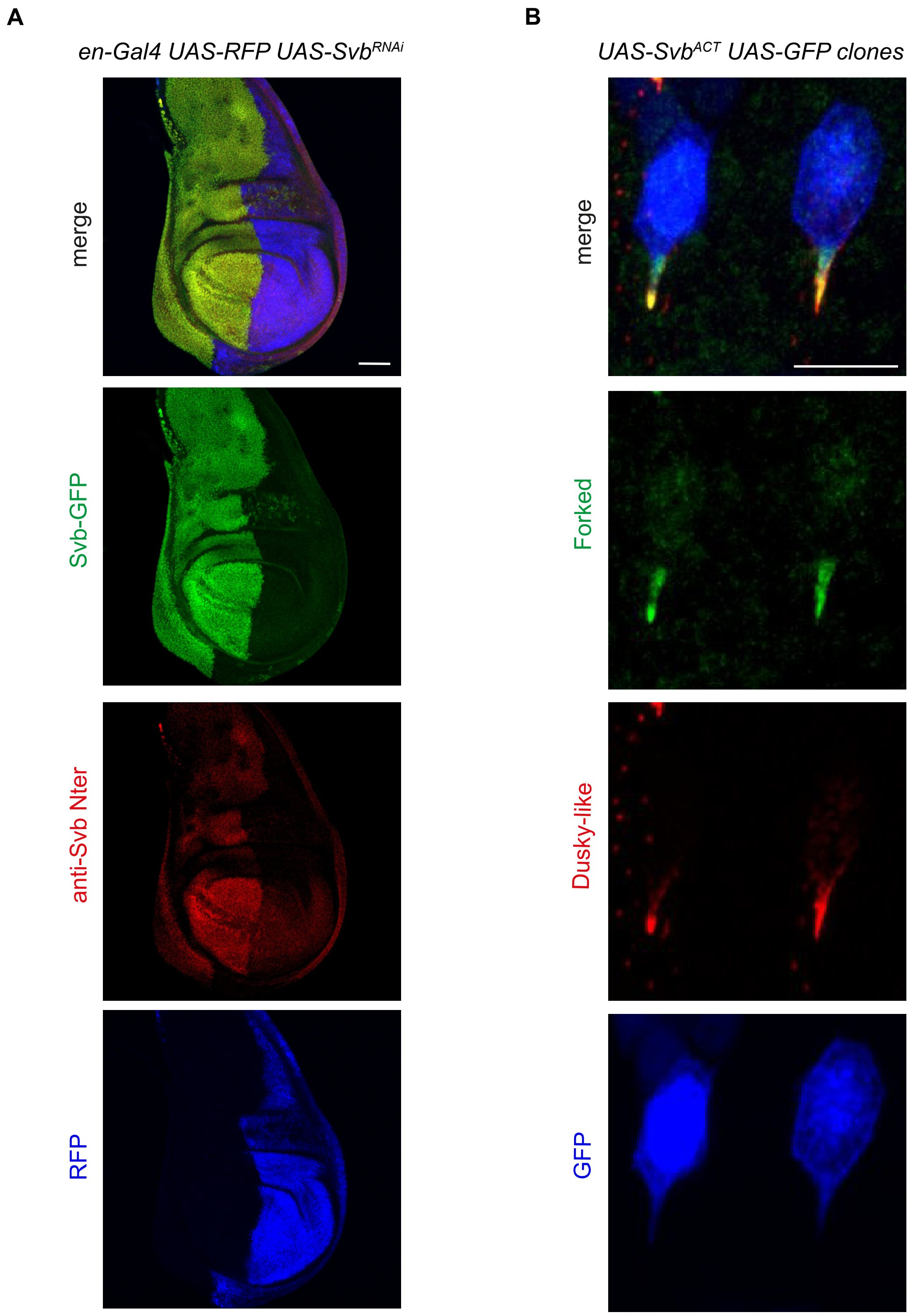

Fig S1. Shavenbaby expression and molecular targets, related to Figures 1 and 2

(A) Confocal images (projection) of a wing disc during L3 stage, when epithelial cells proliferate, in which *svb* was knocked-down by RNAi, driven by *en-Gal4* in the posterior domain as revealed by RFP (blue). The *svb::GFP* knock-in line (green) reveals both Svb^{REP} and Svb^{ACT} protein isoforms, while Svb^{REP} is specifically detected using an antibody recognizing the N-term repressor domain of Svb (see Figure 1). (B) Confocal images of Svb^{ACT} clones revealed by the co-expression of GFP (blue), and stained for Forked (green) and Dusky-like (red), two direct transcriptional targets of Svb. Clones were observed in the notum at 20 h APF. 37 clones were analysed on 4 nota. Scale bars: 50µm in A and 10µm in B.

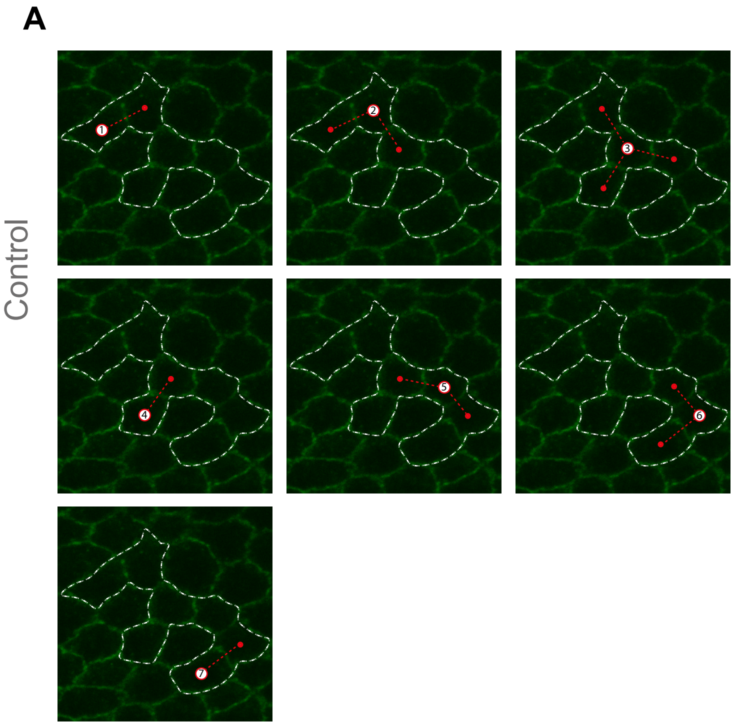

A’

control clone	
cell ID	nb of interactions
1	1
2	2
3	3
4	1
5	2
6	2
7	1
Average nb of interactions	**1,7**

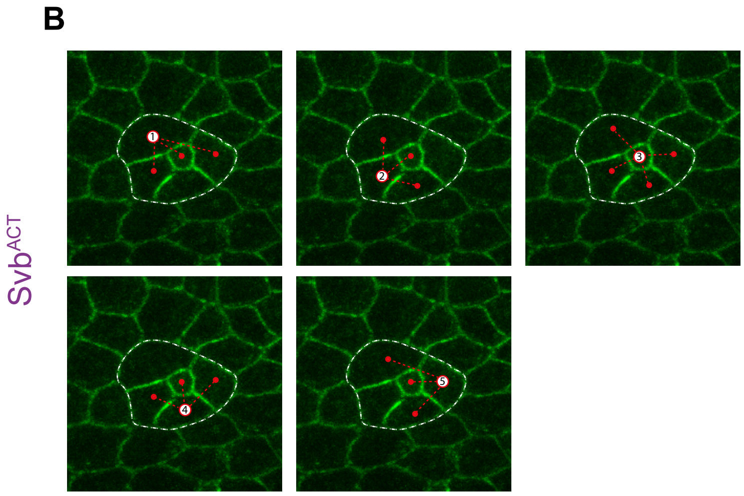

B’

Svb^{ACT} clone	
cell ID	nb of interactions
1	3
2	3
3	4
4	3
5	3
Average nb of interactions	**3,2**

Fig S2. Illustration of the method used to calculate the connectivity index, related to Figure 3

(A, B) Confocal images of control (A) or Svb^{ACT} (B) clones stained for DE-Cadherin (green). White dots show the clonal cells, each cell of the clone was analysed individually. The overlayed number indicates cell ID. Cells that share homotypic junctions with the cell of interest are indicated by red dots. (A’, B’) For each cell of the clones shown in A and B, the number of interactions with other clonal cells is summarized in the table and the average number of interactions is calculated for each clone.

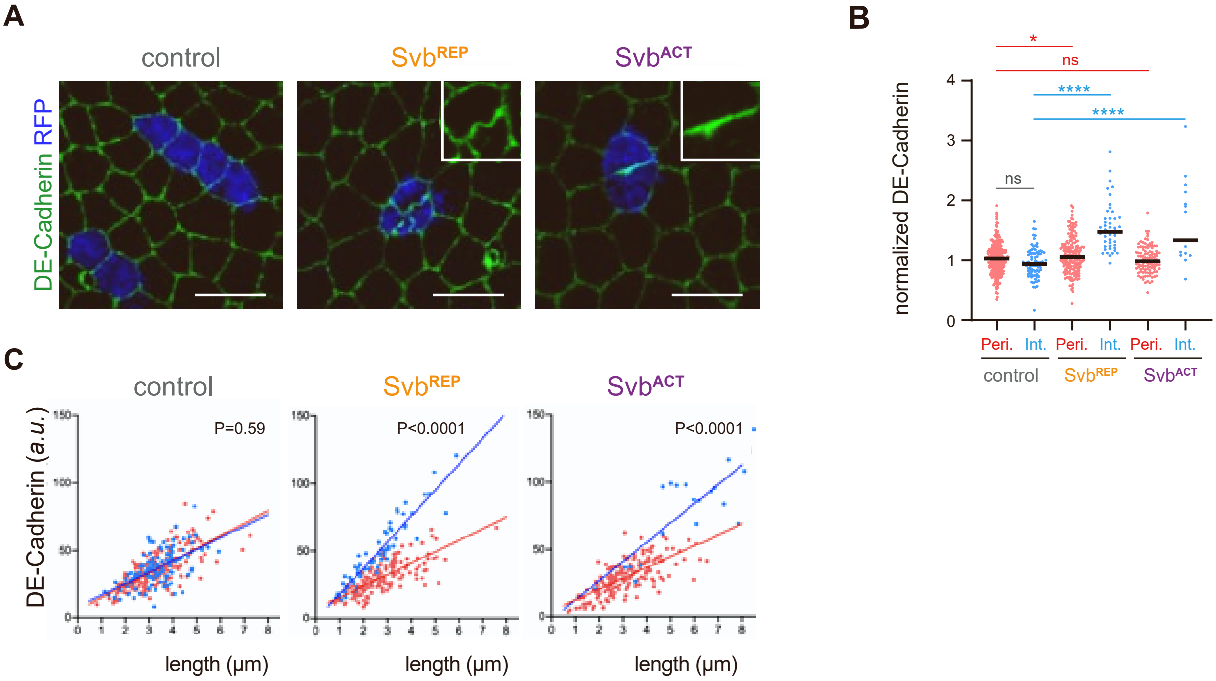

Fig S3. Enrichment of E-Cadherin in homotypical junctions of heterochronous cells, related to Figure 4

(A) Confocal images of control, Svb^{REP} or Svb^{ACT} clones at 38h APF, with adherens junctions revealed by DE-Cadherin staining (green). Clonal RFP-positive cells appear in blue. Close-ups on the internal junctions of the clones are shown. Scale bar: 10µm. (B) Quantifications of normalized E-Cadherin intensity for peripheral (red) and internal (blue) junctions of the clone in control, Svb^{REP} or Svb^{ACT} contexts. Data are from 17 nota with 377 peripheral and 89 internal junctions in controls; 15 nota/182 peripheral and 48 internal junctions for Svb^{REP} clones; 11 nota/111 peripheral and 15 internal junctions for Svb^{ACT} clones. Black bold lines represent medians; P-values are calculated from Mann-Whitney tests, each condition was compared to control junctions. ns $P>0.05$; * $P<0.05$; **** $P<0.0001$. (C) The graph plots raw E-Cadherin intensity against the length of peripheral (red) and internal (blue) junctions, in control, Svb^{REP} or Svb^{ACT} clones. For controls, n=198 peripheral and 108 internal junctions; for Svb^{REP}, n=155 peripheral and 61 internal junctions; for Svb^{ACT}, n=187 peripheral and 20 internal junctions. P-values are the probability that the slopes of peripheral (red) *vs* internal (blue) linear regressions are identical.

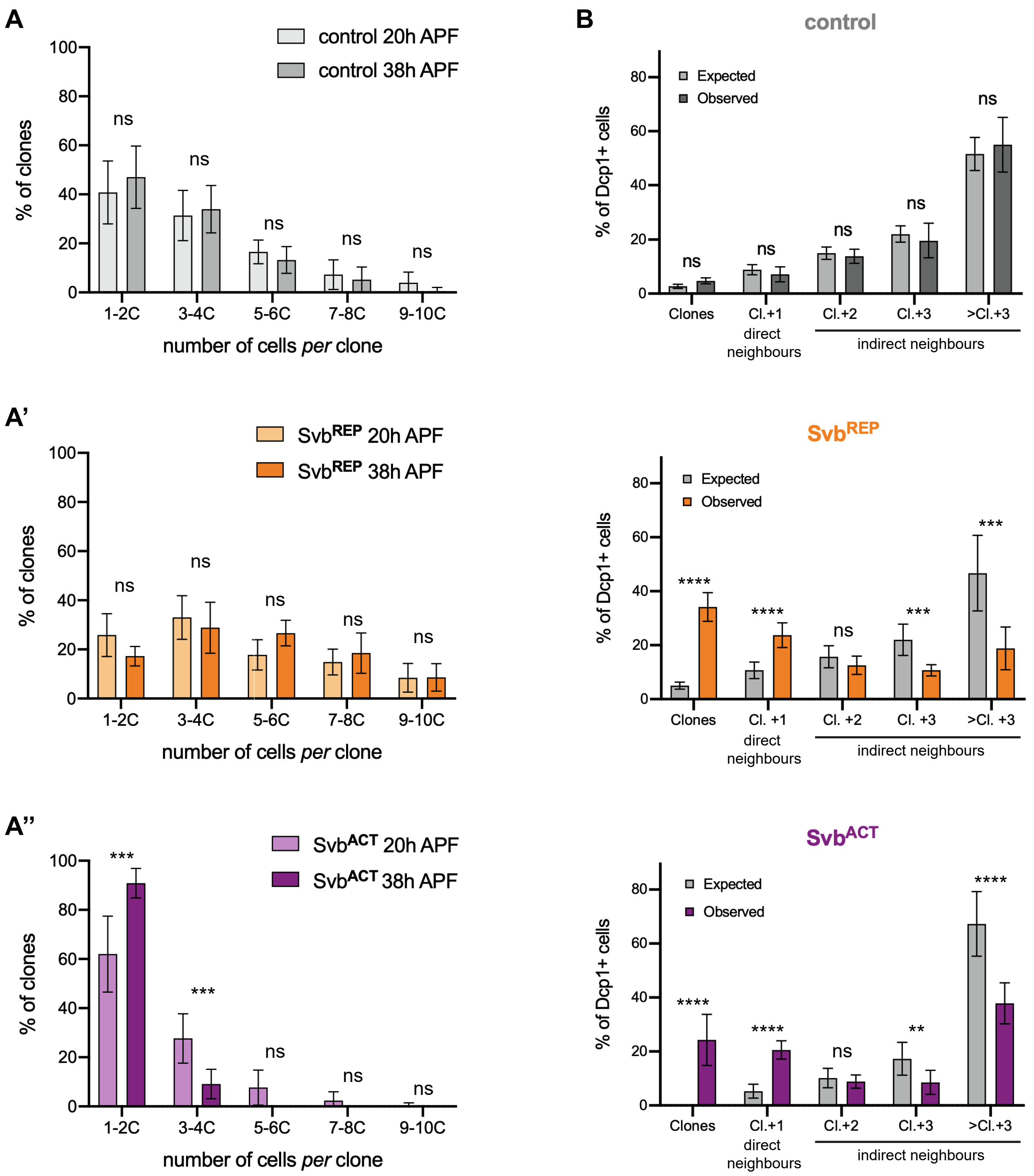

Fig S4. Elimination of clonal cells and their direct neighbours, related to Figure 7

(A-A'') Graphs showing the number of cells *per* clone at 20h APF *vs* 38h APF, in controls (A, grey), Svb^{REP} (A', orange) or Svb^{ACT} (A'', purple) conditions. N=321 clones analysed at 20h APF and 175 at 38h APF in controls; 301 and 150 in Svb^{REP}; 446 and 174 in Svb^{ACT} conditions, respectively. (B) Graphs comparing the percentage of Dcp1-positive cells according to their distance from the clone observed in controls, (grey), Svb^{REP} (orange) or Svb^{ACT} (purple), and corresponding expected values as estimated by a simple probabilistic model (see methods). Cells that are in direct contact to the clone are noted "Cl +1", cells separated from the nearest clone by one, two or more cell diameters are noted "Cl+2", "Cl+3" and ">Cl+3", respectively. Data are from 7 nota with 231 clonal cells and 321 Dcp1+ cells in controls, 7 nota with 1536 clonal and 463 Dcp1+ cells in Svb^{REP}, 7 nota with 324 clonal and 306 Dcp1+ cells in Svb^{ACT} conditions. (A, B) Individual P-values were from t tests, using a False Discovery Rate approach (Q=1%) and two stage step-up method. ns P>0.05; ** P<0.01; *** P<0.001; **** P<0.0001.

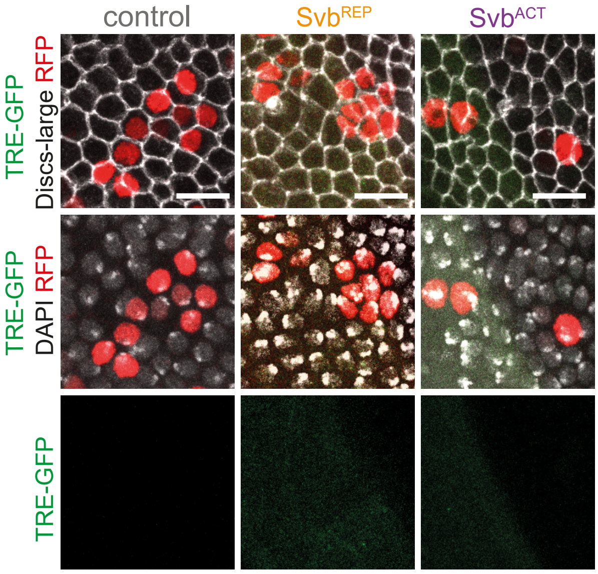

Fig S5. Heterochrony does not activate the JNK signalling pathway in the notum, related to Figure 7

Confocal images of nota from flies expressing TRE-GFP, at 24h APF. TRE-GFP (green) is a reporter of the activation of the JNK pathway. Control, Svb^{REP} and Svb^{ACT} clones were revealed by the nuclear RFP (red). Cells contours were revealed by anti-Discs-large staining, nuclei by DAPI. 9 nota/23 clones in control context (expression of *luc* RNAi); 11 nota/34 clones in Svb^{REP} context; 9 nota/29 clones in Svb^{ACT} context. Scale bar: 10µm.

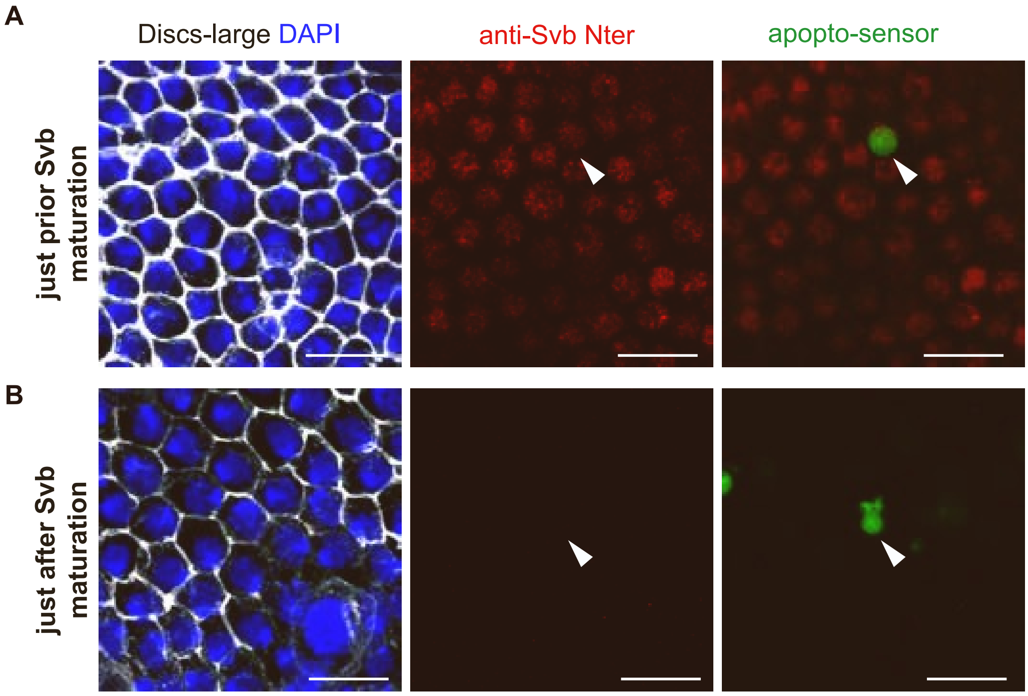

Fig S6. Apoptosis does not affect the status of Svb, related to Figure 8

(A, B) Confocal images of nota taken at the time of Svb processing (38-39h APF) showing either a sample in which cells have not processed Svb yet (A), or a sample in which cells have already processed Svb (B). Discs-large (white) and DAPI (blue) stainings reveal cell contours and nuclei, respectively; anti-Svb N-term (red) and the apopto-sensor (green) mark Svb^{REP} and apoptotic cells, respectively. Arrowheads point to GC3Ai apopto-sensor positive cells. Note that apoptotic cells exhibit similar Svb^{REP} signal than their neighbours. Scale bars: 10µm.
